# The development of an herbal material quality control strategy considering the effects of manufacturing processes

**DOI:** 10.1186/s13020-019-0262-9

**Published:** 2019-09-24

**Authors:** Jingjing Pan, Siyuan He, Jiayao Zheng, Jingyuan Shao, Ning Li, Yunqi Gong, Xingchu Gong

**Affiliations:** 1 Pharmaceutical Informatics Institute. College of Pharmaceutical Sciences, Zhejiang University, Hangzhou, 310058 China; 2 Kunming Pharmaceutical Group Co., Ltd., Kunming, 650100 Yunnan China

**Keywords:** Quality by design, Definitive screening design, *Panax notoginseng*, Quality control strategy

## Abstract

**Background:**

Quality by design (QbD) is an advanced drug quality control concept that has been gradually implemented in the optimization of manufacturing processes of Chinese medicines. However, the variation of Chinese medicinal material quality has rarely been considered in published works. Because manufacturing processes may lower the variation introduced through different batches of materials, a material quality control strategy should be developed considering the influences of manufacturing processes.

**Methods:**

In this work, the processes of extraction, concentration, water precipitation, and chromatography for notoginseng total saponin (NTS) production were investigated while considering *Panax notoginseng* quality variation as a sample. Ten process parameters were studied simultaneously using a definitive screening design. After the process critical quality attributes (CQAs) were determined, critical process parameters (CPPs) and critical material attributes (CMAs) were identified simultaneously. Then, models utilizing the CMAs, CPPs, and process CQAs were developed. The design space was then calculated using a Monte Carlo simulation method with an acceptable probability of 0.90. A material quality control strategy considering the influences of manufacturing processes was proposed.

**Results:**

The ginsenoside Rd purity and total saponin purity in the eluate were identified as process CQAs. The ethanol solution concentration used for extraction, the ethanol solution concentration used for elution, and elution time were identified as CPPs. The extractable dry matter content of *Panax notoginseng* was one of the CMAs. The extractable contents of notoginsenoside R_1_, ginsenoside Rg_1_, ginsenoside Rb_1_, and ginsenoside Rd were the other CMAs. The inequalities implemented to discriminate the high quality and low quality of *Panax notoginseng* were developed according to the NTS standard of the Xuesaitong injection. Low quality *Panax notoginseng* should not be released for NTS production. High quality *Panax notoginseng* can be treated with feasible manufacturing processing parameters. Verification experiments were carried out successfully for 2 batches of high quality *Panax notoginseng.*

**Conclusions:**

In this work, a quality control strategy for herbal materials was developed considering the matching of process characteristics and material quality attributes. This strategy is promising for application to other Chinese medicines.

## Background

Quality by design (QbD) is an advanced drug quality control concept systematically described in Dr. Yu’s work [1]. Knowledge-based management and risk-based management are required in the application of the QbD concept [2]. Recently, the QbD concept was successfully implemented in the optimization of manufacturing processes of Chinese medicines [3] such as extraction [4], precipitation [5], chromatography [6], decoloration [7], drying [8, 9], granulation [10], and so on.

In these works, critical process parameters (CPPs) were identified by risk analysis or statistical analysis [11]. The relationships among CPPs, critical material attributes (CMAs), and process critical quality attributes (CQAs) were investigated using response surface designs [12], such as central composite design or Box-Behnken design. After data collection, quadratic models were usually built [13]. The design space was then calculated with the overlapping method or methods considering the probability to meet all process CQA limits [14]. A control strategy for process parameters was developed, such as the recommendation of normal operation ranges of CPPs [15] and the feedforward control of CPPs [16]. However, the control strategy of material was rarely considered.

The variation of material attributes was considered to be the main source of drug quality variance for Chinese medicines [17]. A set of proper manufacturing processes of Chinese medicines may reduce the variations introduced by materials. Therefore, the development of a material quality control strategy should consider the influences of manufacturing processes.

Wang et al. used central composite circumscribed design to simultaneously study the influences of materials and process parameters [18]. However, the variety of raw materials was very limited. Ye et al. used fixed process parameters to treat different batches of *Salvia miltiorrhiza* to obtain the disodium salt of Salvianolic acid B [19]. The quality standards of water extracts, which were the upstream intermediate, were set to ensure high purity Salvianolic acid B salt preparation [19]. However, the standards of *Salvia miltiorrhiza* were not developed. Therefore, a new method to develop a material control strategy that considers the effects of manufacturing processes is still highly required.

**Fig. 1:**
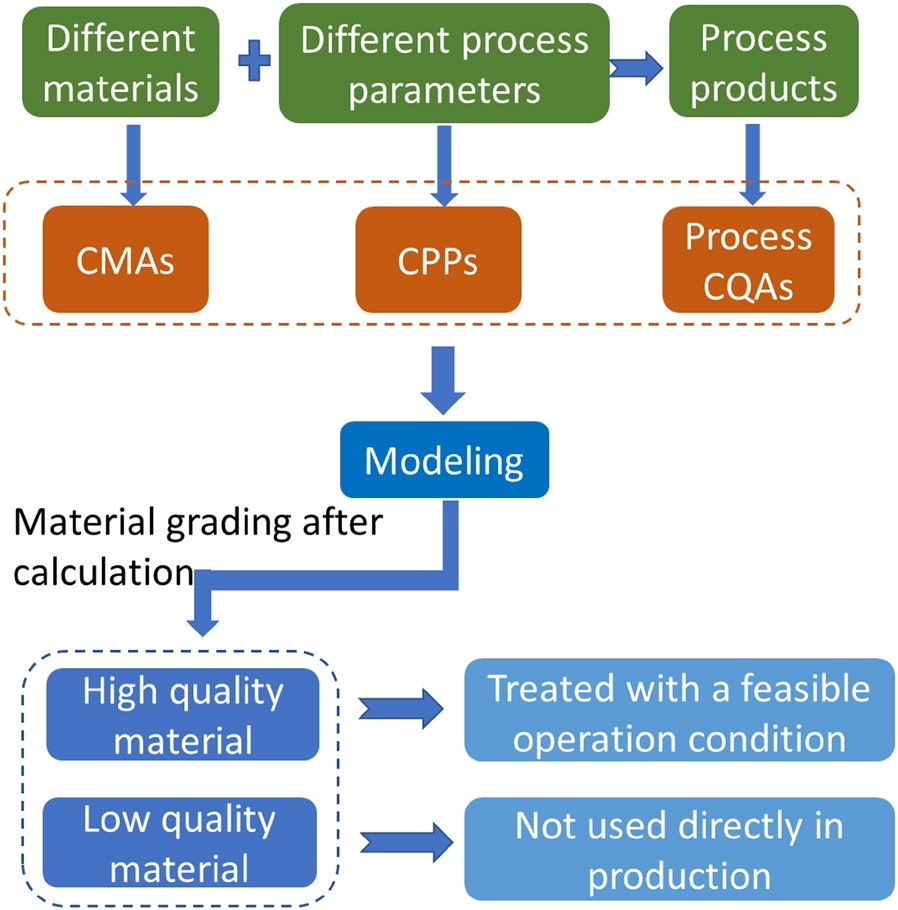
Schematic diagram of the procedures for developing the material control strategy

Notoginseng total saponins (NTS), the material in the Xuesaitong injection, is a kind of herbal extract made from *Panax notoginseng* [20–22]. It is prepared with extraction, concentration, water precipitation, chromatography, decoloration, and drying [23, 24]. In this work, extractable saponin content and extractable dry matter content were considered as potential CMAs of *Panax notoginseng*. Definitive screening design was used to investigate the four processes of extraction, concentration, water precipitation, and chromatography simultaneously. Different materials were used in the experiments. After process CQAs were determined, CPPs and CMAs were identified simultaneously. Then, models implementing CMAs, CPPs, and process CQAs were developed. The design space was then calculated. A material control strategy considering the matching of manufacturing processes and materials was proposed. High quality and low quality *Panax notoginseng* can be discriminated by inequalities and can thus be processed differently. Verification experiments were carried out for high quality *Panax notoginseng*. Figure 1 is a schematic diagram of the development of the material control strategy.

## Methods

### Materials and chemicals

Twenty batches of *Panax notoginseng* were collected from Yunnan Province of China. HPD-100 resin was purchased from Cangzhou Bon Adsorber Technology Co., Ltd. (Cangzhou, China). Reference substances of notoginsenoside R_1_, ginsenoside Rg_1_, ginsenoside Re, ginsenoside Rb_1_, and ginsenoside Rd were purchased from Shanghai Winherb Medical Technology Co., Ltd. (Shanghai, China). Acetonitrile (HPLC grade) was purchased from Merck (Darmstadt, Germany). Deionized water was prepared by an academic water purification system (Milli-Q, Milford, MA, USA). Ethanol (analytical grade) was purchased from Shanghai Lingfeng Chemical Reagent Co., Ltd. (Shanghai, China), and Sinophram Chemical Reagent Co., Ltd. (Shanghai, China).

### Procedures

#### Quality characterization of *Panax notoginseng*

To characterize the material attributes, 100 g of *Panax notoginseng* was pulverized and reflux-extracted with 600 mL of an 80% (v/v) ethanol solution twice. The extraction time of each extraction was 6 h. The two extracts were obtained by filtration and then combined. Then, the concentrations of saponins and dry matter in the combined extract were analyzed. The extractable dry matter content (EDM_PN_) of *Panax notoginseng* was defined as Eq. 1.

1 $$ EDM_{PN} = \frac{{M_{EX} \times DM_{EX} }}{{M_{PN} }} $$

where M and DM refer to the mass and dry matter concentration, respectively; the subscripts EX and PN refer to the combined extracts and *Panax notoginseng*, respectively. The extractable saponin content (ES_PN_) of *Panax notoginseng* was defined as Eq. 2.

2 $$ ES_{PN,i} = \frac{{M_{EX} \times SC_{EX,i} }}{{M_{PN} }} $$

where SC refers to the saponin content; the subscript i where i is an integer value from 1 to 5 represents notoginsenoside R_1_, ginsenoside Rg_1_, ginsenoside Re, ginsenoside Rb_1_, and ginsenoside Rd, respectively. The extractable dry matter content and extractable saponin content were considered as potential CMAs of *Panax notoginseng* in this work.

#### Extraction, concentration, and water precipitation processes

*Panax notoginseng* (100 g) was pulverized and reflux-extracted with an ethanol solution twice. After extraction, the extracts were filtered and combined. The extracts were then concentrated under reduced pressure. The volume of concentrated extracts was controlled. Then, water was added to the concentrated extracts under magnetic stirring (85-1, Hangzhou Instrument Motor Co. Ltd., China). The mixtures were settled at room temperature for 1 h after stirring for 10 min. The supernatants of the water precipitation samples were collected by vacuum filtration. The five parameters of the ethanol solution concentration used for extraction, extraction time, ethanol solution volume, volume of concentrated extract, and water addition amount for water precipitation were investigated. Their coded and uncoded levels in the experiments are listed in Table 1.

**Table 1:** Coded and uncoded values of parameters

Process	Parameters	Symbols	Coded values		
			− 1	0	1
Ethanol extraction	Ethanol solution concentration for extraction (v/v %)	X_1_	80	85	90
	Extraction time (h)	X_2_	6.0	8.0	10.0
	Ethanol solution volume (mL)	X_3_	600	800	1000
Concentration	Volume of concentrated extract (mL)	X_4_	100	150	200
Water precipitation	Water added for water precipitation (mL)	X_5_	300	400	500
Column chromatography	Sampling flow rate (BV/h)	X_6_	1.0	1.5	2.0
	Washing flow rate (BV/h)	X_7_	1.0	1.5	2.0
	Water volume (BV)	X_8_	1.0	2.0	3.0
	Ethanol solution concentration for elution (v/v %)	X_9_	55	60	65
	Elution time (min)	X_10_	120	150	180

#### Column chromatography process

A chromatography column was filled with 720 mL of HPD-100 microporous resin. The inner diameter of the column was 6 cm. Supernatants collected after water precipitation were passed through the column. A defined volume of water was then used to wash the column. After that, a volume of ethanol solution was used to elute the saponins. The elution flow rate was fixed at 1.0 BV/h. The eluate was collected for 30 min during elution in all experiments. The resin was regenerated with a 90% (v/v) ethanol solution at a flowrate of 1.5 BV/h for 80 min and then with water at 2 BV/h for 1.5 h. In the chromatography process, the five parameters of the sampling flow rate, washing flow rate, water volume, ethanol solution concentration used for elution, and elution time were investigated. Their coded and uncoded levels in the experiments can be seen in Table 1.

#### Experimental design

In this work, a total of 10 parameters belonging to the 4 different processes of reflux extraction, concentration, water precipitation, and column chromatography were investigated simultaneously. To reduce the number of experiments, definitive screening design was used to investigate the influences of these manufacturing process parameters. Quadratic models can also be developed based on the definitive screening designed experiment results [25]. Many process parameters can also be studied in a very small number of experiments [25]. The experimental conditions are listed in Table 2. To study the effects of raw materials, different batches of *Panax notoginseng* were used in these experiments, as shown in Table 2.

**Table 2:** Conditions of definitive screening designed experiments

No.	*Panax notoginseng*	X_1_ (v/v %)	X_2_ (h)	X_3_ (mL)	X_4_ (mL)	X_5_ (mL)	X_6_ (BV/h)	X_7_ (BV/h)	X_8_ (BV)	X_9_ (v/v %)	X_10_ (min)
1	PN12	85	10.0	1000	200	500	2.0	2.0	3.0	65	180
2	PN7	85	6.0	600	100	300	1.0	1.0	1.0	55	120
3	PN10	90	8.0	1000	100	500	2.0	1.0	1.0	55	120
4	PN2	80	8.0	600	200	300	1.0	2.0	3.0	65	180
5	PN15	90	10.0	800	200	300	2.0	2.0	1.0	55	120
6	PN3	80	6.0	800	100	500	1.0	1.0	3.0	65	180
7	PN11	90	6.0	1000	150	500	1.0	2.0	3.0	55	120
8	PN10	80	10.0	600	150	300	2.0	1.0	1.0	65	180
9	PN8	90	10.0	600	200	400	2.0	1.0	3.0	65	120
10	PN11	80	6.0	1000	100	400	1.0	2.0	1.0	55	180
11	PN12	90	10.0	1000	100	500	1.5	2.0	1.0	65	180
12	PN14	80	6.0	600	200	300	1.5	1.0	3.0	55	120
13	PN4	90	6.0	1000	200	300	2.0	1.5	3.0	55	180
14	PN9	80	10.0	600	100	500	1.0	1.5	1.0	65	120
15	PN6	90	6.0	600	200	500	1.0	2.0	2.0	65	120
16	PN8	80	10.0	1000	100	300	2.0	1.0	2.0	55	180
17	PN2	90	6.0	600	100	500	2.0	1.0	3.0	60	180
18	PN16	80	10.0	1000	200	300	1.0	2.0	1.0	60	120
19	PN5	90	6.0	600	100	300	2.0	2.0	1.0	65	150
20	PN6	80	10.0	1000	200	500	1.0	1.0	3.0	55	150
21	PN1	90	10.0	600	100	300	1.0	2.0	3.0	55	180
22	PN4	80	6.0	1000	200	500	2.0	1.0	1.0	65	120
23	PN16	90	10.0	1000	100	300	1.0	1.0	3.0	65	120
24	PN15	80	6.0	600	200	500	2.0	2.0	1.0	55	180
25	PN13	90	6.0	1000	200	300	1.0	1.0	1.0	65	180
26	PN7	80	10.0	600	100	500	2.0	2.0	3.0	55	120
27	PN3	90	10.0	600	200	500	1.0	1.0	1.0	55	180
28	PN13	80	6.0	1000	100	300	2.0	2.0	3.0	65	120
29	PN9	85	8.0	800	150	400	1.5	1.5	2.0	60	150
30	PN14	85	8.0	800	150	400	1.5	1.5	2.0	60	150
31	PN5	85	8.0	800	150	400	1.5	1.5	2.0	60	150
32	PN1	85	8.0	800	150	400	1.5	1.5	2.0	60	150

### Analytical methods

Dry matter content was measured using a precision electronic balance (AB204-N, Mettler Toledo Shanghai Co., Ltd). The samples were dried at 105 °C in an oven (DZF-6050, Shanghai Jing Hong Laboratory Instrument Co., Ltd.) for 3 h and then kept in a desiccator for 0.5 h. The quantitative analysis method for the determination of the saponin concentrations was reported in previous work [26]. The method was performed on a Waters Acquity UPLC system (Waters, Milford, MA, USA) using a Waters CSH C18 column (50 mm × 2.1 mm i.d., 1.7 μm) at 40 °C. The mobile phase consisted of 0.01% formic acid–water (A) and 0.01% formic acid–acetonitrile (B) using the following gradient program: 0–6 min, 18–20% B; 6–6.8 min, 20–30% B; 6.8–11 min, 30–35% B; 11–17 min, 35–90% B; and 17–25 min, 90% B. The flowrate was 0.35 mL/min, and the volume of the sample injection was 5 μL. The detector wavelength was set to 203 nm.

### Data processing

The total saponin concentration (TSC) value in the extract or eluate was calculated with Eq. 3.

3 $$ TSC = \sum\limits_{i = 1}^{5} {SC_{i}^{{}} } $$

The total saponin yield (TSY) was defined as Eq. (4).

4 $$ TSY = \frac{{M_{EL} \times TSC_{EL} }}{{M_{PN} }} $$

where the subscript EL refers to the eluate.

Dry matter yield (DMY) was calculated with Eq. (5).

5 $$ DMY = \frac{{M_{EL} \times DMC_{EL} }}{{M_{PN} }} $$

Saponin purity (SP) values were calculated using Eq. 6.

6 $$ SP_{i} = \frac{{C_{EL,i}^{{}} }}{{DM_{EL} }} \times \, 100\% $$

The total saponin purity (TSP) value in the eluate was calculated with Eq. 7.

7 $$ TSP = \sum\limits_{i = 1}^{5} {SP_{i}^{{}} } $$

Design expert V8.0.6.1 (State-Ease Inc. MN) was used to analyze the results of the definitive screening designed experiments. To identify CPPs and CMAs, Eq. (8) was used to model process parameters, quality attributes of *Panax notoginseng*, and process CQAs.

8 $$ Y = a_{0} + \sum\limits_{i = 1}^{11} {b_{i} X_{i} } + \sum\limits_{k = 1}^{7} {c_{k} Z_{k} } $$

where Y is a process CQA, X_i_ (i = 1–10) represents a process parameter, Z_k_ (k = 1–6) represents a material attribute, a_0_ is a constant, and b_i_ (i = 1–10) and c_k_ (k = 1–6) are regression coefficients. Stepwise regression was used to simplify the models. Insignificant variables were removed by stepwise regression. The significance levels for adding terms and removing terms were both set to 0.05. Any term remaining in the model was considered to represent a CPP or a CMA.

Then, CPPs’ interactive sand square terms were added to establish predictive models for process CQAs, as seen in Eq. (9).

9 $$ Y = a_{0} + \sum\limits_{i = 1}^{n} {b_{i} X_{i} } + \sum\limits_{i = 1}^{n} {b_{ii} X_{i}^{2} } + \sum\limits_{i = 1}^{n - 1} {\sum\limits_{j = i + 1}^{n} {b_{ij} X_{i} X_{j} } + } \sum\limits_{k = 1}^{m} {c_{k} Z_{k} } $$

where n is the number of CPPs and m is the number of CMAs. Stepwise regression was also performed as mentioned above.

A Monte Carlo method simulating the random error of experimental values was performed to calculate the design space. The detailed calculation processes are described in previous work [27]. A brief description is given as follows: the experimental results were assumed to be normally distributed. The mean value of the normal distribution was assumed to be the measured response value. The relative standard deviation (RSD) of the normal distribution was fixed at 0.04. Random values representing experimental values were then created by the Monte Carlo simulation according to the normal distribution. These random values were modeled by Eq. 9. After each simulation, a new equation for a process CQA was obtained. The new equations developed from the Monte Carlo simulation values were used to calculate the predicted results. The probability of meeting all process CQA goals was then calculated based on the model prediction results. The design space was defined with a probability higher than 0.90. The simulation was performed 1000 times to obtain a probability-based design space. All calculations were carried out using MATLAB (R2016a, Version 9.0, The Math Works Inc., USA).

## Results

### Material attributes

The quality attributes of different *Panax notoginseng* batches are shown in Table 3. For the extractable saponin contents, the ginsenoside Rg_1_ content was the highest, and the ginsenoside Re content was the lowest. For most materials, more than 100 mg/g *Panax notoginseng* of saponins can be extracted. The extractable dry matter contents were between 185 and 387 mg/g *Panax notoginseng.*

**Table 3:** Quality attributes of different batches of *Panax notoginseng*

No.	Extractable saponin content (mg/g *Panax notoginseng*)					Extractable dry matter content (mg/g *Panax notoginseng*) (Z_6_)
	Notoginsenoside R_1_(Z_1_)	Ginsenoside Rg_1_(Z_2_)	Ginsenoside Re(Z_3_)	Ginsenoside Rb_1_(Z_4_)	Ginsenoside Rd(Z_5_)	
PN1	15.9	59.2	7.35	52.9	14.0	363
PN2	8.42	32.1	3.21	30.4	6.99	185
PN3	14.3	53.9	7.74	47.5	11.2	355
PN4	7.18	33.4	4.64	31.3	5.17	216
PN5	14.5	52.7	7.79	51.8	11.0	387
PN6	13.3	52.4	8.37	48.0	11.3	342
PN7	8.31	34.6	4.50	30.4	5.89	192
PN8	10.4	48.6	6.95	43.9	7.89	363
PN9	13.4	49.4	8.27	45.5	10.4	337
PN10	13.3	47.5	7.64	46.6	9.79	356
PN11	8.99	38.5	4.41	29.7	5.03	224
PN12	12.4	46.8	7.83	42.7	9.19	357
PN13	14.5	48.3	6.33	46.3	11.2	304
PN14	8.04	36.0	4.38	29.0	5.07	197
PN15	15.1	51.7	6.42	48.3	13.1	341
PN16	14.8	55.7	7.10	51.3	14.2	316

### Process CQA identification

After analyzing the eluates collected after column chromatography, the process quality attributes were calculated and were determined to be saponin purities, total saponin yield, and dry matter yield in the eluates. As shown in Table 4, the total saponin yield in the eluate was between 66.9 and 146 mg/g *Panax notoginseng.* Due to the removal of impurities, the dry matter yield was between 76.3 and 175 mg/g *Panax notoginseng,* which was significantly lower than the extractable dry matter of corresponding materials. Total saponin purity increased significantly to between 77.85 and 91.02%. The purity of ginsenoside Rd was between 3.15 and 8.58%.

**Table 4:** The quality attributes of the manufacturing processes

No.	Saponin purity (%)					Total saponin purity (%)	Dry matter yield (mg/g *Panax notoginseng*)	Total saponin yield (mg/g *Panax notoginseng*)
	Notoginsenoside R_1_	Ginsenoside Rg_1_	Ginsenoside Re	Ginsenoside Rb_1_	Ginsenoside Rd			
1	8.65	33.33	5.23	30.19	6.60	83.99	144	121
2	9.16	39.41	5.37	30.07	3.64	87.65	76.3	66.9
3	9.45	35.33	5.19	30.40	4.75	85.12	142	121
4	8.62	33.27	3.38	32.51	7.68	85.46	90.2	77.1
5	10.00	35.67	5.32	29.96	5.56	86.51	140	121
6	8.85	35.77	4.44	30.77	7.28	87.10	153	133
7	10.59	42.97	5.20	28.91	3.34	91.01	79.4	72.3
8	7.60	29.95	4.51	29.48	6.32	77.85	166	130
9	7.11	34.97	4.81	30.12	5.70	82.70	125	103
10	8.75	38.88	4.79	30.38	4.06	86.85	94.8	82.3
11	8.20	33.85	4.99	29.43	6.50	82.96	126	104
12	9.74	43.44	5.28	29.85	3.15	91.47	80.7	73.8
13	8.29	37.19	5.38	32.94	4.74	88.53	82.1	72.6
14	8.52	32.22	4.84	29.92	6.70	82.20	143	118
15	8.23	33.54	5.30	30.39	7.11	84.57	117	99.0
16	7.38	33.32	4.88	30.87	4.82	81.26	148	121
17	8.88	33.91	4.08	30.93	7.43	85.23	82.5	70.3
18	8.54	32.32	4.40	28.94	6.79	80.99	170	137
19	8.52	31.38	4.60	29.94	6.57	81.01	138	111
20	8.15	33.32	5.49	29.14	5.28	81.38	153	125
21	9.22	35.01	4.81	30.52	7.02	86.57	162	140
22	7.23	34.68	4.92	32.14	5.18	84.16	97.1	81.7
23	8.75	33.11	4.18	29.52	7.71	83.27	166	138
24	9.70	33.40	4.43	29.54	6.57	83.64	152	127
25	9.61	32.78	4.13	31.22	8.58	86.31	137	119
26	9.08	38.66	5.61	30.37	3.90	87.61	91.0	79.8
27	8.23	33.98	5.64	30.30	6.21	84.37	137	116
28	9.35	31.69	4.06	30.55	7.26	82.92	146	121
29	8.86	33.54	5.24	30.49	6.65	84.79	153	130
30	9.57	40.20	5.20	32.82	5.23	93.02	86.8	80.7
31	8.26	30.95	4.79	29.16	5.91	79.07	168	133
32	8.85	33.01	4.45	29.75	7.47	83.53	175	146

The dry matter yield mainly affects the economic benefits of pharmaceutical enterprises. By dividing saponin yield and dry matter yield, saponin purity can be calculated. Saponin purity represented the chemical composition of the process product. Therefore, saponin purity was considered a potential process for CQAs.

In the quality standards of the Xuesaitong injection, the purity of notoginsenoside R_1_, ginsenoside Rg_1_, ginsenoside Re, and ginsenoside Rb_1_ in the NTS should not be lower than 6%, 28%, 2.5%, and 26%, respectively. In Table 4, the minimum purities of the above four saponins were 7.11%, 29.95%, 3.38%, and 28.91%, respectively. This means that these four process quality attributes easily attained their standards. Therefore, they were not considered as process CQAs. However, the minimum obtained Rd purity and total saponin purity were lower than that of their standards and were 3.5% and 85%, respectively. Therefore, Rd purity and total saponin purity were used as process CQAs.

### The identification of CMAs and CPPs

The linear regression results of Eq. 8 and the corresponding ANOVA results are shown in Table 5. The P value of each model was lower than 0.0001, indicating that the models were significant. The determination coefficients (R^2^) of all the models were higher than 0.70, which indicated that most of the data variances can be explained. It also indicated that the major influencing factors were probably included in the models. According to the P values, the ethanol solution concentration used for extraction, the ethanol solution concentration used for elution, and elution time were selected as CPPs. The extractable contents of notoginsenoside R_1_, ginsenoside Rg_1_, ginsenoside Rb_1_, and ginsenoside Rd of *Panax notoginseng* were CMAs. The extractable dry matter content of *Panax notoginseng* was also a CMA.

**Table 5:** Regression coefficients, R^2^ values and ANOVA results for the multiple linear regression models

Process parameters	Y_1_(Rd purity)		Y_2_(Total saponin purity)	
	Coefficient	p value	Coefficient	p value
Constant	− 0.12		0.76	
X_1_	3.382 × 10^−4^	0.0394*	2.203 × 10^−3^	0.0056**
X_9_	1.824 × 10^−3^	< 0.0001**		
X_10_	1.869 × 10^−4^	< 0.0001**		
Z_1_			0.0103	0.0143*
Z_2_			5.291 × 10^−3^	0.0023**
Z_4_			− 0.0111	< 0.0001**
Z_5_	3.971 × 10^−3^	< 0.0001**		
Z_6_	− 8.607 × 10^−5^	< 0.0001**		
Model p value	< 0.0001		< 0.0001	
R^2^	0.9348		0.7460	
${\text{R}}^{ 2}_{\text{adj}}$	0.9223		0.7083	

* p < 0.05

** p < 0.01

### The effects of CMAs and CPPs

Compared with Eq. 8, the nonlinear effects and interaction effects of CPPs were considered in Eq. 9, which was evaluated in this work. However, after stepwise regression, the terms representing nonlinear effects and interaction effects were all taken out of the models. This result indicated that only linear effects of CPPs were significant.

According to the regression coefficient values in Table 5, higher ethanol concentrations in the extraction process resulted in higher ginsenoside Rd purity and total saponin purity. As the ethanol solution concentration used for elution and the elution time increased, the ginsenoside Rd purity increased. For CMAs, higher ginsenoside Rd and lower extractable dry matter content both led to a higher ginsenoside Rd purity in the eluate. Higher notoginsenoside R_1_, higher ginsenoside Rg_1_, and lower ginsenoside Rb_1_ purities all resulted in a higher purity of total saponins.

### Design space development

**Fig. 2:**
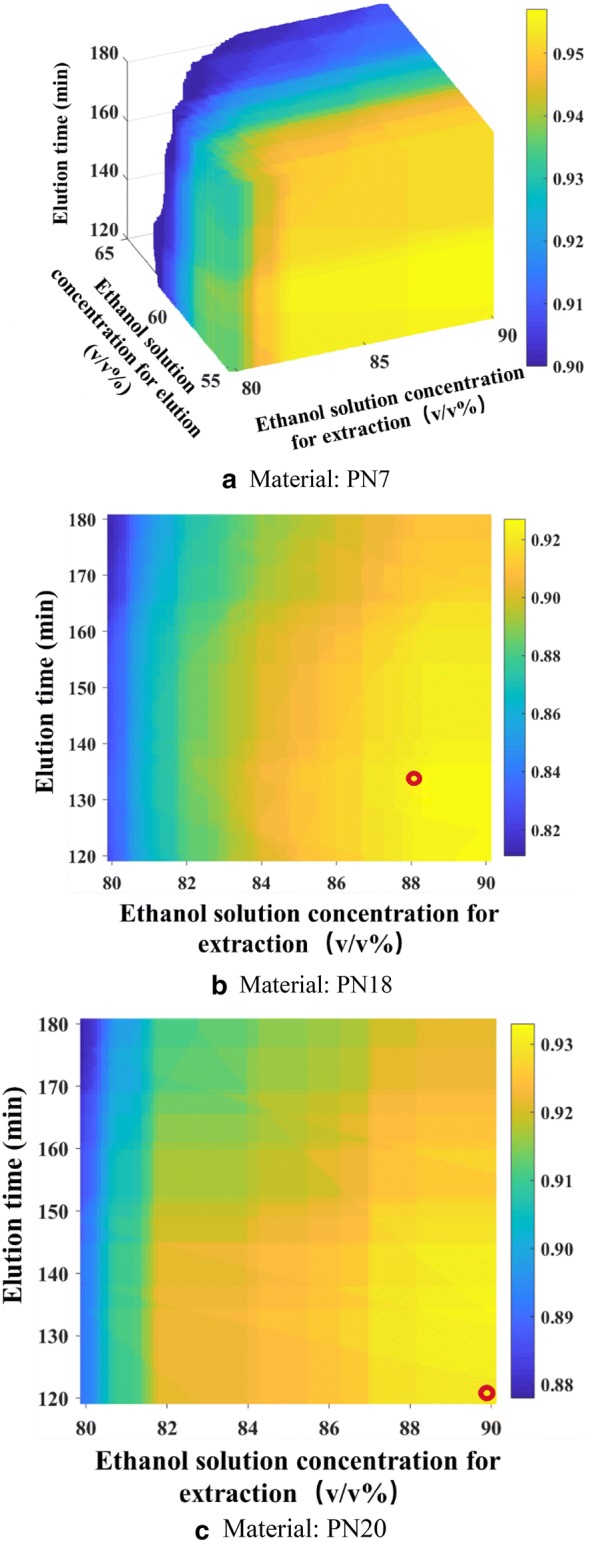
Three-dimensional design space and verification points (color bar refers to the probability of attaining the process CQA criteria; ○, verification points). **a** Material: PN7, **b** material: PN18, **c** material: PN20

The lower limits of Rd purity and total saponin purity were set according to the NTS standards of the Xuesaitong injection, which were 3.5% and 85%, respectively. Then, the design space was calculated, as shown in Fig. 2a and Additional file 1: Table S1. The design space was an irregular region.

### Control strategy of *Panax notoginseng*

To prepare satisfactory eluates, Inequalities 1 should be satisfied for Rd purity and total saponin purity.

1 $$ \left\{ \begin{aligned} & b_{1}^{1} X_{1} + b_{9}^{1} X_{9} + b_{10}^{1} X_{10} + c_{5}^{1} Z_{5} + c_{6}^{1} Z_{6} + a_{0}^{1} \ge 3.5\% \hfill \\ & b_{1}^{2} X_{1} + c_{1}^{2} Z_{1} + c_{2}^{2} Z_{2} + c_{4}^{2} Z_{4} + a_{0}^{2} \ge 85.0\% \hfill \\ \end{aligned} \right. $$

where superscript 1 and 2 refer to Rd purity and total saponin purity, respectively. The values of regression coefficients in Inequalities 1 can be found in Table 5. In principle, different batches of *Panax notoginseng* can be divided into two grades according to Inequalities 1. If the CMAs of a batch of *Panax notoginseng* meet Inequalities 1, the batch of *Panax notoginseng* is considered to be acceptable material if the process parameters can be varied within the investigated ranges. In contrast, if Inequalities 1 is unattainable, the batch of *Panax notoginseng* is considered to be unacceptable and cannot be released for NTS production. For a batch of acceptable *Panax notoginseng*, feasible process parameters can be chosen after calculation or selected from Additional file 1: Table S1.

However, in the current traditional Chinese medicine TCM industry, raw materials are usually treated with a set of fixed manufacturing process parameters because of the limitation of regulations. In this case, the quality control of raw material should be stricter.

For example, the manufacturing process parameters were fixed as follows: the ethanol solution concentration for extraction was 88%, the extraction time was 6 h, the ethanol solution addition was 6.72 mL/g *Panax notoginseng*, the volume of concentrated extract was 1.31 mL/g *Panax notoginseng*, and the water addition for water precipitation was 3.73 mL/g *Panax notoginseng*. The sampling flow rate was 1.7 BV/h, the washing sampling flow rate was 1.8 BV/h, the washing volume was 2.0 BV, the ethanol solution concentration for elution was 56%, and the elution time was 133 min.

Inequalities 1 can then be simplified to Inequalities 2.

2 $$ \left\{ \begin{aligned} &397Z_{5} - 8.61Z_{6} + 730 \ge 0 \hfill \\ &10.3Z_{1} + 5.291Z_{2} - 11.1Z_{4} + 108.3 \ge 0 \hfill \\ \end{aligned} \right. $$

Then, Inequalities 2 can be used to divide different batches of *Panax notoginseng* into two grades. A batch of *Panax notoginseng* with CMAs meeting Inequalities 2, this batch of *Panax notoginseng* is considered to be high quality material for the current parameter fixing process. If a batch of *Panax notoginseng* with CMAs does not meet Inequalities 2, it is considered a low-quality material and should not be released for NTS production.

### Examples of material quality control and verification experiments

The CMAs of 4 batches of *Panax notoginseng* were measured and are shown in Table 6. According to Inequalities (1), *Panax notoginseng* of PN17 and PN19 were low quality *Panax notoginseng*, and they should not be directly used in production. However, PN17 and PN19 were probably qualified materials because their saponin contents met the standards in Chinese Pharmacopeia (1st Section, 2015 Edition). If they were qualified materials, they can also be used in the existing manufacturing processes after proper mixing with other qualified batches of *Panax notoginseng* to form a new batch.

**Table 6:** Material attributes of *Panax notoginseng*

	Notoginsenoside R_1_(Z_1_)	Ginsenoside Rg_1_(Z_2_)	Ginsenoside Rb_1_(Z_4_)	Ginsenoside Rd(Z_5_)		
PN17	6.55	37.1	35.9	7.19	242	Low quality
PN18	8.05	38.7	34.0	7.41	241	High quality
PN19	8.67	51.6	46.8	7.42	312	Low quality
PN20	9.09	38.7	32.3	6.43	227	High quality

PN18 and PN20 were high quality *Panax notoginseng* batches. The verification experiment conditions were as follows: for PN18, the fixed process parameters mentioned in “Control strategy of *Panax notoginseng*” section were used. For PN 20, the ethanol concentration was 90% (v/v), the extraction time was 10 h, the ethanol solution addition was 6.0 mL/g *Panax notoginseng*, the volume of the concentrate was 1.5 mL/g *Panax notoginseng*, the water addition for water precipitation was 3 mL/g *Panax notoginseng*, the sampling flow rate was 1.0 BV/h, the water volume was 1 BV, the ethanol solution concentration for elution was 60% (v/v), and the elution time was 120 min. The results are listed in Table 7. All the indices met the standards of NTS of the Xuesaitong injection. The prediction results were close to the experimental results, indicating that the models were accurate.

**Table 7:** Results of verification experiments

No.	No.	Saponin purity (%)					Total saponin purity (%)
		Notoginsenoside R_1_	Ginsenoside Rg_1_	Ginsenoside Re	Ginsenoside Rb_1_	Ginsenoside Rd	
PN18	1	11.29	37.44	5.26	30.46	5.06	89.51
	2	11.47	37.03	5.90	32.00	5.53	91.92
	3	12.29	38.50	6.24	31.74	5.23	94.00
	Predicted values	–	–	–	–	4.70	86.50
PN20	1	10.04	41	4.47	30.7	5.47	91.69
	2	10.51	43	4.61	31.1	5.2	94.39
	3	9.77	40.1	4.72	30.7	5.24	90.58
	Predicted values	–	–	–	–	4.83	89.81

## Discussion

### The selection of potential CMAs

Potential CMAs can be physical, chemical, and biological features of *Panax notoginseng.* Considering that NTS is mainly composed of five saponins, their contents in *Panax notoginseng* should be potential CMAs. The method to analyze saponin contents in *Panax notoginseng* can be found in Chinese Pharmacopeia (1st Section, 2015 Edition) and other publications [28–30]. *Panax notoginseng* needs to be pulverized into a powder and extracted with methanol or a methanol solution in these works. However, *Panax notoginseng* will not be pulverized as finely in industry processes. The extraction solvent is an ethanol solution instead of a methanol solution. This means that the saponins extracted in an industrial process may be less than that in an analytical laboratory for the same material. Therefore, extractable saponin content was used in this work as a potential CMA.

In addition to the abovementioned five saponins, there are many other components in *Panax notoginseng*. However, only the components that can be extracted by the ethanol solution may affect the composition of NTS. Therefore, extractable dry matter was used as another potential CMA in this work. The usage of extractable dry matter also avoided the difficulties in measuring low content components in *Panax notoginseng*. Extractable dry matter and extractable saponin content were measured after extraction experiments with conditions similar to industrial conditions.

### The reduction of CPP number

In this work, three CPPs were identified for four processes: the ethanol solution concentration used for extraction, the ethanol solution concentration used for elution, and the elution time. If these processes were studied separately, as in previous works [6, 13, 31, 32], there were more than 10 CPPs. These results indicated that CPP number can be reduced if several processes are studied simultaneously. A lower CPP number means that the design of the process control strategy can be simplified. There is no CPP in water precipitation and concentration, which means that these processes are not critical processes. This conclusion also agreed well with Zhong et al.’s work [33] and He’s work [34].

### The relationship of material control strategy and regulations

In the current industry, manufacturing process parameters are fixed despite the variation of herbal material quality on most occasions. However, in principle, process parameters can vary within their ranges declared by drug regulatory authorities. However, if the declared ranges of manufacturing process parameters are very narrow, the variation of process parameters will be difficult. Although the application for changing a manufacturing process is an option, the cost for Type II and Type III change is usually not economically feasible. Therefore, strict control of the quality of medicinal materials becomes the better option. An example of determining material quality standards for the parameter fixing process is given in “Control strategy of *Panax notoginseng*” section.

### The disadvantages of the present method

Equation 9 was used to model CMAs, CPPs, and process CQAs [35]. However, the interaction between CMAs and CPPs was not considered. The nonlinear effects of CMAs were also ignored. After stepwise regression, the interaction between CPPs was not found. A main reason is that the experimental number was not large enough after using the current experimental design.

Extractable dry matter content and five extractable saponin contents were considered potential CMAs. However, some CMAs may be missing. Although different batches of *Panax notoginseng* were used, the representativeness of material quality may still be insufficient.

## Conclusion

In this work, the manufacturing processes of extraction, concentration, water precipitation, and chromatography for NTS production were studied with the consideration of *Panax notoginseng* quality variation. A definitive screening design was used for the study of 10 process parameters. Different materials were used in the experiments. Extractable saponin contents and extractable dry matter content were considered potential CMAs. The purity of ginsenoside Rd and the purity of total saponins were selected as process CQAs. The ethanol solution concentration used for extraction, the ethanol solution concentration used for elution, and elution time were selected as CPPs. Extractable dry matter content and extractable contents of notoginsenoside R_1_, ginsenoside Rg_1_, ginsenoside Rb_1_, and ginsenoside Rd were CMAs. Quantitative models of CPPs, CMAs, and process CQAs were obtained. The design space was then calculated using a Monte Carlo simulation method with an acceptable probability of 0.90. High quality and low quality *Panax notoginseng* can be discriminated with Inequalities 1. Low quality *Panax notoginseng* should not be directly used in production. High-quality *Panax notoginseng* can be treated with feasible process parameters. Verification experiments were carried out for high quality *Panax notoginseng.* The prediction results agreed well with the experimental results, indicating that the models were accurate. In this work, a quality control strategy of raw *Panax notoginseng* material was developed considering the matching of manufacturing process characteristics and material quality attributes. A similar strategy can also be applied to other Chinese medicines.

## Supplementary information

**Additional file 1: Table S1.** Design space and calculated probability.

## Data Availability

All data generated or analyzed during this study are included in this published article and its supplementary information files.

## Abbreviations

NTS: Notoginseng total saponins
CMAs: critical material attributes
CQAs: critical quality attributes
CPPs: critical process parameters
QbD: quality by design
DSD: definitive screening design
EDM: extractable dry matter content
ES: extractable saponin content
M: mass
DM: dry matter concentration
SC: saponin content
TSC: total saponin concentration
TSY: total saponin yield
DMY: dry matter yield
SP: saponin purity
TSP: total saponin purity

## Superscript

EX: combined extracts
PN: *Panax notoginseng*
EL: eluate
